# The importance of search strategy for finding targets in open terrain

**DOI:** 10.1186/s41235-017-0049-4

**Published:** 2017-02-20

**Authors:** Charlotte A. Riggs, Katherine Cornes, Hayward J. Godwin, Simon P. Liversedge, Richard Guest, Nick Donnelly

**Affiliations:** 1 University of Southampton, School of Psychology, Highfield, Southampton, Hampshire SO17 1BJ UK; 2 University of Kent, School of Engineering and Digital Arts, Canterbury, Kent CT2 7NT UK

**Keywords:** Visual search, Large-scale search, Search strategy, Paired searching, Foraging, Multiple targets

## Abstract

A number of real-world search tasks (i.e. police search, detection of improvised explosive devices (IEDs)) require searchers to search exhaustively across open ground. In the present study, we simulated this problem by asking individuals (Experiments 1a and 1b) and dyads (Experiment 2) to search for coin targets pseudo-randomly located in a bounded area of open grassland terrain. In Experiment 1a, accuracy, search time, and the route used to search an area were measured. Participants tended to use an ‘S’-shaped pattern with a common width of search lane. Increased accuracy was associated with slower, but also variable, search speed, though only when participants moved along the length (as opposed to across the width) of the search area. Experiment 1b varied the number of targets available within the bounded search area and in doing so varied target prevalence and density. The results confirmed that the route taken in Experiment 1a generalizes across variations in target prevalence/density. In Experiment 2, accuracy, search time, and the search strategy used by dyads was measured. While dyads were more accurate than individuals, dyads that opted to conduct two independent searches were more accurate than those who opted to split the search space. The implications of these results for individuals and dyads when searching for targets in open space are discussed.

## Significance

The current work was inspired by an effort to inform and improve training and procedures in two search tasks: first, for police teams combing open ground for clues to a crime; and second, the detection of improvised explosive devices (IEDs) for soldiers on patrol in theatre. These tasks are related in that both require an exhaustive search and are often performed by teams as well as individuals. Despite being an important class of search problem, currently little is known about how searchers maximize the chances of finding targets in these situations. The present study is a first effort to understand this important class of search problem. It does so by recording route maps and information about participants’ speed of movement, as well as accuracy and response times, to describe how searches were conducted. These searches were conducted by individuals (in relation to search route and speed) and dyads (in relation to decisions about splitting search over space). While only a first step, a thorough understanding of what underlies accurate searching in this class of search problem will have profound implications for those involved in training and developing procedures for search teams involved in finding targets situated in open spaces.

## Background

What do we know about how exhaustively police search teams comb open ground for clues to a crime or how soldiers patrolling high-risk routes search for IEDs? The unfortunate answer is that, at present, we understand very little. With a few exceptions (e.g. Foulsham, Chapman, Nasiopoulos, & Kingstone, 2014; Gilchrist, North, & Hood, 2001; Jiang, Won, Swallow, & Mussack, 2014), previous studies on human search ability have primarily explored visual search (see Eckstein, 2011 for a recent review) or foraging (Cain, Vul, Clark, & Mitroff, 2012; Wolfe, 2013) using experimental stimuli presented on computer screens.

There are three major differences that should be noted between computer-based tasks and the task of searching for small targets placed in open space. First, the spatial scale is sufficiently different that small targets in open spaces are unlikely to be detected through pre-attentive or attentive vision alone (e.g. Treisman & Gelade, 1980; Wolfe, 2007; Wolfe, Cave, & Franzel, 1989) but will also require head and body movement (i.e. physical foraging behavior). Second, unlike terminating search following the detection of a single target (Tuddenham, 1962) or when the rewards of continuing foraging in one area are less than those that might follow from moving to a new area (Cain et al., 2012; Wolfe, 2013), searchers must attempt to search spatial locations exhaustively (by which we mean try their best to search as many possible target locations as they can). Third, if we consider real-world situations, such as police searching for clues to a crime or soldiers searching for IEDs, searchers have to consider all potential instances of all potential target types (Godwin et al., 2015), rather than finding well-defined targets matching a simple template. The present study is a first effort in trying to understand how exhaustive search is in these types of task. In this study, our focus is limited to understanding the search strategies that maximize the exhaustiveness of search. In the present case, we explore this issue by examining search for an unknown number of targets, which are distributed across an area of open space.

Search strategies have previously been explored in studies of eye movements, in terms of fixation patterns in active vision tasks (e.g. Gilchrist & Harvey, 2006; Keech & Resca, 2010). They have also been explored in terms of the approach undertaken by typical individuals and brain-damaged patients when performing computerized versions of pencil and paper cancellation tasks (Dalmaijer, Van der Stigchel, Nijboer, Cornelissen, & Husain, 2014; Donnelly et al., 1999). Despite differences in tasks and goals across these studies, a common conclusion is that detecting targets is better when search follows specific, structured paths.

The utility of using systematic paths for search is also apparent in mathematical models aimed at optimizing real-world search. Search Theory (Koopman, 1946, 1980) was developed in World War II to facilitate maritime search, rescue, and detection operations. The theory is an application of probability theory to search such that the likelihood of targets being found at specific locations can be computed, enabling searching of areas likely to contain targets to be prioritized. On the basis of these calculations, a search path is defined over which a plane or ship can pass in search of targets, with an improved chance of detecting them. Search Theory is still being developed, with recent applications for search and rescue on land (Koester et al., 2014; Robe & Frost, 2002). Search Theory can be utilized for simple through to complex search scenarios. In the simplest of cases, the likelihood of targets appearing within a search area might follow a uniform distribution. More complex cases must take account of Gaussian distribution of probabilities for factors such as initial target locations, drift patterns caused by air and water currents, and perhaps a desire for targets to remain hidden.

Considering the simplest of cases, optimal paths should minimize the distance of the route that allows all locations to be searched (using body, head, and eye movements) without making revisits. Evidence can be found in support of humans using such a strategy in Gilchrist et al. (2001). They explored whether the principles from visual search studies could be applied to large-scale search tasks. Using a task where all potential target locations were set in a regular array and clearly visible on the ground, they examined how the search for marble targets hidden in film canisters was conducted. The important result was that, in contrast to visual search, rechecking locations was rare, with participants making fewer revisits to canisters they had already searched. Gilchrist et al. (2001) suggested that there was a higher cost (in terms of the physical effort to cross the room) associated with forgetting in their task relative to standard visual search and that the increased effort required to search led to a higher likelihood of participants remembering searched locations. A similar result was reported by Smith, Hood, and Gilchrist (2008) in the same large-scale experimental setting and also by Ruddle and Lessels (2006). In all these tasks, minimizing re-checking must have involved determining a route through the search array.

Gilchrist et al. (2001) used a regular, visible search array (although targets were hidden in film canisters and required checking, these canisters were clearly laid out on the floor), but what happens when targets are hard to find and potential target locations are not arranged in a regular fashion? Critical to understanding how humans might conduct effective target search in such circumstances is what is meant by trying to search exhaustively across space. Consider the case where a given search area is wider than that which can be searched in a single pass. Within Search Theory (Koopman, 1946, 1980), the distance to the left and right of each location that still allows target detection defines an area known as the effective search width (ESW). The technical limit of sensors (human vision, radar, etc.) determines the width of the ESW and therefore how close together neighboring passes of a single path should be. With respect to humans, we refer to the width of the search corridor rather than the ESW as human search is not deterministic. Whether a particular setting of search width is considered effective for target search is dependent on knowing the accuracy of target detection within a search corridor.

For humans, without making head and eye movements to the left and right, the search width will be determined by the limits of foveal and parafoveal vision to discriminate targets. However, head and eye movements can be made to overcome the limits of foveal vision so long as they are calibrated with the forward speed of travel (along the search corridor being ‘swept’). If the speed of sweeping along a search path is too fast to allow search of adjoining spaces to the left, right, and center, then search will be incomplete. We conclude that the likelihood of search being conducted in a fine-grained and exhaustive manner will depend on a range of factors. In the absence of existing data, it seems likely that search will be subject to the strategic decisions made by searchers regarding systematicity of search and the trade-off of their chosen width of search corridor for sweeping, with their forward speed of search. Experiment 1a was designed to reveal evidence of individuals using a search strategy (in terms of the forward speed of search, the width of the corridor being swept and consistency of a chosen strategy) and to assess the influence of this strategy on search accuracy and overall speed.

## Experiment 1a

In Experiment 1a, individual participants searched for an unknown number of coin targets placed in open grassland terrain. We were interested in how search strategy was related to both target detection accuracy and search time for the task. The grassland was 75 m^2^ in size. Participants searched the open space in any manner they chose and for however long they wished to search. Accuracy was measured as the number of targets detected and search time was calculated as the time from when the participants started the task until they told the experimenters they had finished.

It is important to understand that it was not possible for participants to detect all but very few targets from their starting point. Target detection required moving through the search area. More than the detection of targets per se, or the type of targets searched for, it is the need to conduct an exhaustive search through a search area for targets that are very hard to find that connects this task most directly with that of finding clues to a crime or to the presence of an IED. Analysis of the systematicity of search was enhanced by using data extracted from a Total Station theodolite system. These data allow for visual representation of the routes taken by participants (henceforth ‘route maps’: see Fig. 3). They can also be processed using Fourier analysis to provide some quantitative evidence for general trends apparent when inspecting representations of routes taken by participants. This approach is helpful as participants are free to move in any direction and it captures the underlying spatio-temporal properties of their movements overall.

The Fourier analysis transforms the data from the time domain to the frequency domain and enables calculation of multiple measures derived from the frequency components of movement along the x-axes and y-axes of the search area: (1) The dominant frequency component is an index of the modal speed of movement along each axis. The reciprocal of the dominant frequency component (1/dominant frequency) converts this to seconds and can be used as an approximate measure of participant’s modal time before changing direction; (2) Dividing participant’s overall search time by the modal time before changing direction gives an estimate of the number of changes of direction. Plotting the number of changes of direction along the x-axis against those on the y-axis allows determination of whether participants tended to move systematically along x-axes or y-axes (i.e. left to right or top to bottom) or use a hybrid strategy. Furthermore, by dividing the number of changes of direction by the length of the axis being travelled across provides an estimate of the width of the search corridor used by participants; and finally (3) dividing participants’ modal time before changing direction by the next most commonly occurring time before changing direction indexes variability in speed of searching.

In addition to basic data around search accuracy and time, we show typical route maps and analyze the width of search corridors used, modal movement speed, and variability in speed of movement. We predicted that more accurate search would be reliant on: (1) increased regularity, systematicity of search following a structured path (Dalmaijer et al., 2014; Donnelly et al., 1999; Gilchrist & Harvey, 2006; Keech & Resca, 2010); (2) narrower search corridors (Koopman, 1946, 1980); and (3) slower, more consistently paced movement (Koopman, 1946, 1980).

### Method

#### Participants

Thirty participants (7 men and 23 women, mean age = 23.5 years, SD = 4.73) recruited from the University of Southampton community, with normal or corrected-to-normal color vision, took part in the study for course credit. Participants were screened to ensure visual acuity and normal color vision using Snellen (1862) chart for visual acuity and the Ishihara (1917) color plates. The study was performed in accordance with the Declaration of Helsinki and was approved by the University of Southampton, School of Psychology ethics committee. Informed consent was obtained from all participants.

#### Apparatus

Experimenters used a grid representing the larger-scale search space to record accuracy. When participants found a target, they pointed to the target and informed the experimenter, who then marked off the corresponding target on the grid. Overall search time was recorded using a stopwatch. Search was deemed to have finished once participants reported to the experimenter that they were done.

Participant movement over space and time was recorded using a Total Station theodolite (Leica TPS1200, Heerbrugg, Switzerland). The Total Station used electronic distance measurement technology and an angle-measurement system to calculate the coordinate of an unknown point relative to a known coordinate point. A signal was sent from a fixed recording station to a reflector prism mounted on a 1.8-m staff held by the participants. A coordinate was recorded every 2 s and accuracy was within 3 mm per km of distance (SD = 1.5). The output of time-stamped coordinates was processed using Environmental Systems Research Institute’s ArcGIS software (ESRI, 2011).

#### Stimuli

**Fig. 1:**
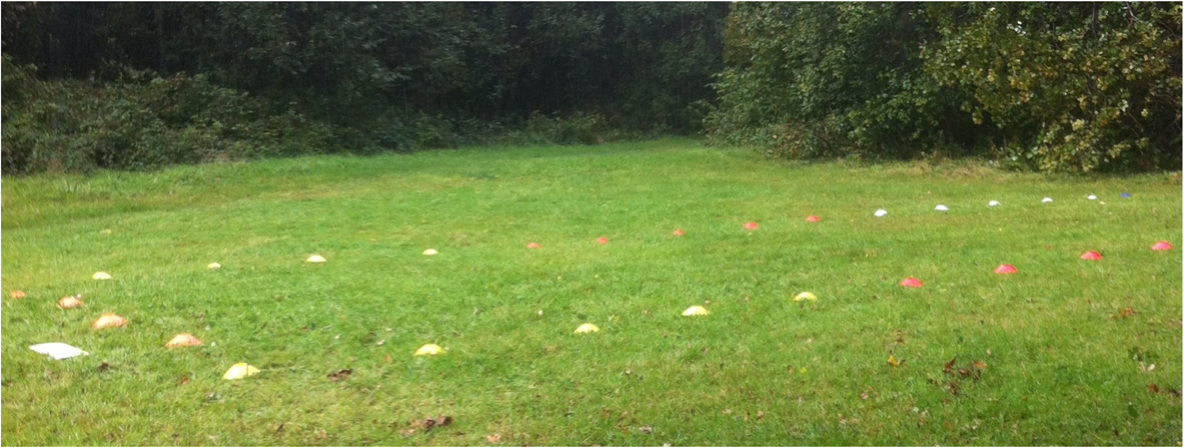
The search grid. An example photograph of the 75 m^2^ grid in which participants searched for coin targets. The perimeter of a 15 × 5 m search area was marked out at 1-m intervals using 40 colored cones

The experiment was conducted on an open space of grassland (see Fig. 1). The perimeter of a 15 × 5 m search area was marked out at 1-m intervals using 40 colored cones. The position of the cones was calibrated with the Total Station prior to testing. The relative positions of cones allowed definition of 75 m^2^ grid cells. Testing took place over four consecutive days. The location of the grid was moved each day to avoid excessive trampling of grass. Across days, the conditions of the grass remained broadly similar.

**Fig. 2:**
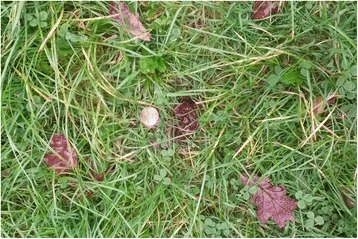
A target. An example photograph of a UK sterling two pence coin target set within the search grid. Note the leaves which occurred naturally and acted as distractors

Twenty-five UK sterling two pence coins were used as targets (see Fig. 2). The two pence coins were of a copper color and were 25 mm in diameter. The coins were all matt rather than shiny in appearance. The coins were placed so that they could be detected from standing height, although they were sufficiently small that detection required active exploration of the search grid.

Coins were distributed within the grid so that five coins were placed pseudo-randomly within each 1-m ‘search lane’ (i.e. five coins were placed along each 1-m search lane across the 5-m width of the grid). On average, there was one coin per 3 m^2^. The targets were placed in the same grid cell locations for all participants. Distractors were not added within the search area but leaves and other natural materials did form naturally occurring distractors and were not removed from the search space.

#### Procedure

Following the screening, participants were given a brief explanation of the Total Station and were instructed to hold the staff upright and close to their body while searching. The staff was lightweight and easy to carry. Participants were not told how many coins could be found but instructed to search until they were confident they had completed their search (i.e. they had found all the targets). Participants were told not to pick coins up but to point and tell the experimenter that a coin had been found. Participants were not penalized for reporting the same coin on more than one occasion (as the task simulates a task where a conservative approach to finding targets is encouraged) but each target was only counted once when calculating response accuracy. Once participants had completed their search, they were asked to give a score on a ten-point scale to indicate how confident they were that they had found all the coins. A higher score implied high confidence while a lower score indicated lower levels of confidence.

### Results

Participants were excluded from data analysis if they failed to detect any coins. While detecting no coins might reflect their best performance, they were removed on the basis that it is impossible to differentiate poor performance from failing to engage with the task. We therefore took a conservative position on removing from analysis. This resulted in the removal of two participants (6.7% of the data) meaning data analysis was conducted on the data from 28 participants. However, the removal of the two participants did not influence the pattern of significance of results. Correlational tests were used to examine if there was a relationship between two variables, simple linear regressions were used to examine whether one variable predicted a second variable, t-tests were used to examine whether the mean scores of two participant groups differed and a Fisher’s exact test was used to test how likely it was that observed distributions were due to chance. All regressions reporting time or frequency use log-transformed data to reduce skew, though this did not, however, affect the underlying pattern of results. For the regressions, significance levels were adjusted for multiple comparisons as regressions compared the same measure across x-axes and y-axes. Only effects reaching a *p* value of 0.025 were considered significant. The statistical package used to analyze the data was R version 3.3.0 (R Core Team, 2016).

#### Behavioral data

Basic measures of accuracy and total search time are presented in Table 1. On average, participants found just under half the available targets, despite spending an average of 7.5 min on the task. On average, participants reported a confidence score of 6.89 on a ten-point scale. On average, the first target was found after 39 s and the last target was found 40 s before terminating search. For each participant, a regression was carried out exploring the linear relationship between the time of finding each target against the ordinal number of that target as found by the participant (i.e. 1^st^, 2^nd^, 3^rd^, etc.). This measure explores whether targets were found consistently throughout search or were found more easily at the beginning than the end of search. The range of adjusted R-squared values was 0.842–0.995 (all *ps* < 0.052) across participants. The result suggests that targets were found at a fixed rate throughout search. Accuracy was predicted by confidence ratings (β_1_ = 2.448, *F*(1,26) = 5.195, *p* = 0.031, adj R^2^ = 0.135): participants who gave a high confidence rating were more accurate in their search.

**Table 1:** Behavioral data

	Accuracy					Total search time (min:s)				
	Experiment 1a	Experiment 1b			Experiment 2	Experiment 1a	Experiment 1b			Experiment 2
	Individuals	5 targets	15 targets	25 targets	Dyads	Individuals	5 targets	15 targets	25 targets	Dyads
Mean	0.45 (0.04)	0.76 (0.19)	0.84 (0.16)	0.80 (0.11)	0.68 (0.13)	07:33 (02:32)	03:51 (02:49)	03:41 (02:00)	03:41 (01:40)	06:15 (04:13)
Minimum	0.12	0.40	0.47	0.52	0.40	02:32	01:19	01:38	01:05	01:13
Maximum	0.80	1	1	0.96	0.92	25:08	07:35	08:32	11:53	18:05

The mean, minimum, and maximum scores for accuracy and total search time for the individuals in Experiment 1a, the individuals in the three target frequency conditions of Experiment 1b and the dyads in Experiment 2

Parentheses indicate Standard Deviation

#### Search strategy

**Fig. 3:**
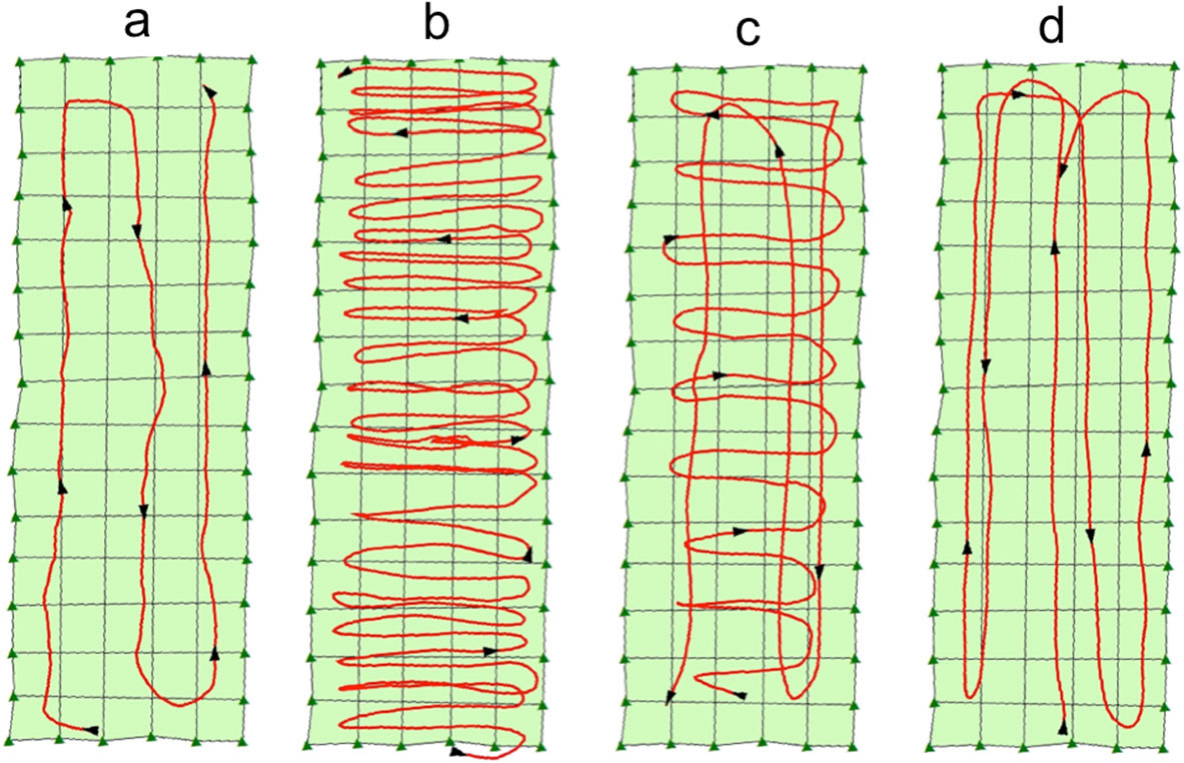
*Route maps* for Experiment 1a. Illustrations of four examples of routes taken when searching. The examples have been chosen to illustrate how the commonly used ‘S’-shape strategy could occur alone or as part of a more complex search, and fixed along the *x- or y- axes* (i.e. searching left to right or top to bottom)

Examples of typical route maps are shown in Fig. 3. Visual inspection of these route maps reveals some commonalities across participants. These commonalities were explored using Fourier analysis.

#### Systematicity of search path

To quantify regularity of search path, a Fourier transformation was carried out for each participant and along both the x- and y- axes (see Donnelly et al., 1999). Our first prediction was that search would be more accurate when participants used a regular, systematic search strategy following a structured path. To explore this prediction, we examined the number of changes of direction (calculated by dividing participant’s overall search time by the modal time before changing direction).

**Fig. 4:**
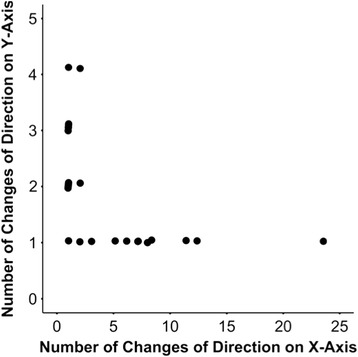
The number of changes of direction on each axis. On the basis of the Fourier analysis, we computed participants’ modal time before changing direction on each axis, using their dominant frequency component. Dividing participant’s overall search time by the modal time before changing direction provided an estimate of the number of changes of direction. Figure 4 shows the relationship between the number of changes of direction on the *x- and y- axes*. Note, non-logged data are plotted throughout

The number of changes of direction was plotted across both axes (see Fig. 4). High values on one axis were associated with low values on the other axis (*r*(26) = –0.558, *p* = 0.002). Participants turning top to bottom tended to search along fewer, longer corridors and participants turning left to right tending to search along more, shorter corridors. These data are consistent with all participants searching systematically, using an ‘S’-shaped route to cover the search area (as shown in Fig. 3). Their fundamental pattern of movement (the ‘S’ shape) was consistent irrespective of whether they primarily moved top to bottom or left to right. The important result is that all participants exhibited regularity in the path taken to search. Given this, it was not possible to explore variations in accuracy as a function of the presence or absence of regularity (as per our first prediction).

#### Width of search corridors

Our second prediction was that search would be more accurate as search width narrowed. This hypothesis was based on the idea that a narrow search width would better facilitate the search of close space using the fine-grained spatial acuity of foveal vision. To explore this prediction, participants were split into two groups – those who primarily moved left to right (*n* = 11) and those who primarily moved top to bottom (*n* = 16). There was one participant who did not fall into either category, conducting a hybrid strategy, and was therefore removed from subsequent analysis. We took the number of changes of direction made by each participant along their dominant axis (i.e. whether they were in the top-to-bottom or left-to-right group), added 1 (to take into account the number of sweeps both up and down, i.e. five turns would mean six sweeps), and divided the length of the axis being travelled across by this figure (i.e. for those in the top-to-bottom group, the length of the axis being travelled across in meters, which was 5, would be divided by the first figure calculated). This normalized the data, as calculating the search width took into account the length of each axis.

**Fig. 5:**
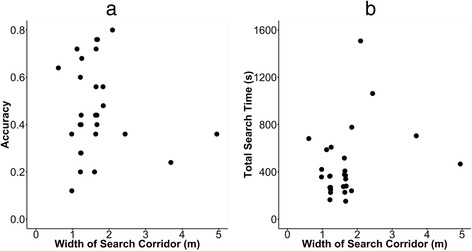
Search width as a predictor for Accuracy (**a**) and Total Search Time (**b**). On the basis of the Fourier analysis, we computed an approximation of participants’ modal time before changing direction on each axis, using their dominant frequency component. Dividing participant’s overall search time by the modal time before changing direction provided an estimate of the number of changes of direction To take into account the number of sweeps both up and down, 1 was added to the number of changes of direction made by each participant along their dominant axis (i.e. whether they were in the left-to-right or top-to-bottom search group) and the length of the axis being travelled across was divided by this figure. This provided a normalized search width in meters (m). Accuracy was the proportion of coins detected and Total Search Time indicates the total amount of time spent searching in seconds (s)

These data are shown in Fig. 5. The striking result is that the search width for the majority of participants lies between 1 and 2 m (alternatively between 50 cm and 1 m to both the left and right of the center). This suggests that there is commonality in search width irrespective of whether participants search top to bottom or left to right across the search grid. Given the limited range of width of search corridors, there is no evidence of search width predicting either accuracy (β_1_ = –0.534, *F*(1,25) = 0.356, *p* = 0.556, adj R^2^ = –0.025) or total search time (β_1_ = 0.446, *F*(1,25) = 2.11, *p* = 0.159, adj R^2^ = 0.041). Irrespective of outcome for accuracy or time, participants searched along their ‘S’-shaped path, using a common search width.

#### Search speed and search speed variability

**Fig. 6:**
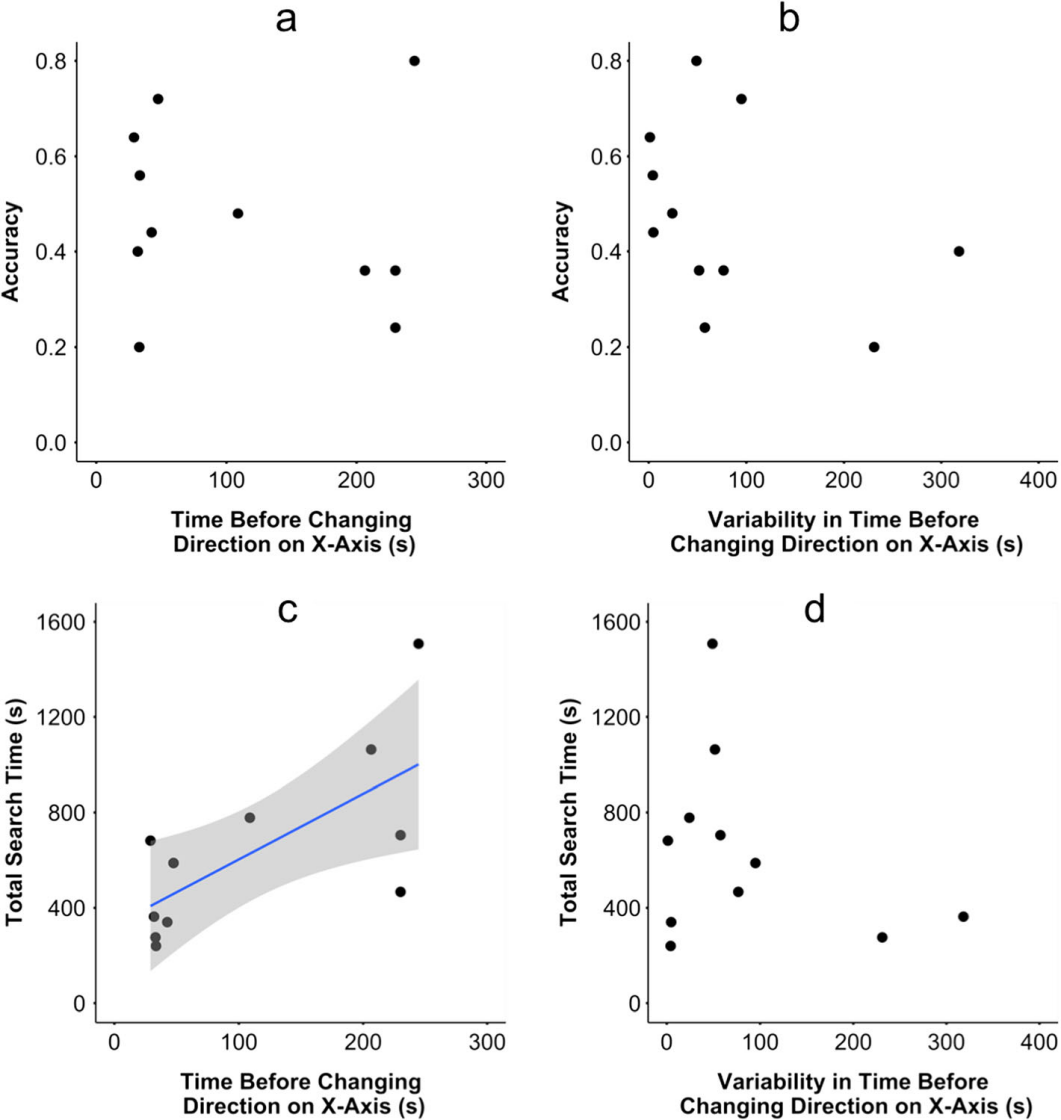
Search speed to predict Accuracy and Total Search Time for left-to-right participants on the *x-axis*. On the basis of the Fourier analysis, we computed an approximation of participants’ modal time before changing direction in seconds (s) on each axis, using their dominant frequency component. By dividing the modal time before changing direction by the next most commonly occurring time before changing direction we computed the variability in time before changing direction in seconds (s). Accuracy was the proportion of coins detected and Total Search Time indicates the total amount of time spent searching in seconds (s). Figure 6 shows the modal time before changing direction (**a**) and the variability in time before changing direction (**b**) to predict Accuracy, plus the modal time before changing direction (**c**) and the variability in time before changing direction (**d**) to predict Total Search time. Note: *lines of fit* are shown when a statistically significant relationship is present

Our third prediction was that search would be more accurate when participants searched with slow, consistently paced movement (Search Theory; Koopman, 1946, 1980). To examine this prediction, we examined the modal time before changing direction on each axis and variability in time before changing direction (by dividing the modal time before changing direction by the next most commonly occurring time before changing direction). For the left-to-right group, accuracy was not predicted by the modal time before changing direction, nor variability in time before changing direction (β_1_ = –0.41, *F*(1,9) = 0.065, *p* = 0.805, adj R^2^ = –0.103; β_1_ = –3.437, *F*(1,9) = 1.478, *p* = 0.255, adj R^2^ = 0.046; see Fig. 6a and b). Total search time was predicted by modal time before changing direction but not variability in time before changing direction (β_1_ = 1.133, *F*(1,9) = 8.712, *p* = 0.016, adj R^2^ = 0.435; β_1_ = 0.094, *F*(1,9) = 0.009, *p* = 0.928, adj R^2^ = –0.11; see Fig. 6c and d).

**Fig. 7:**
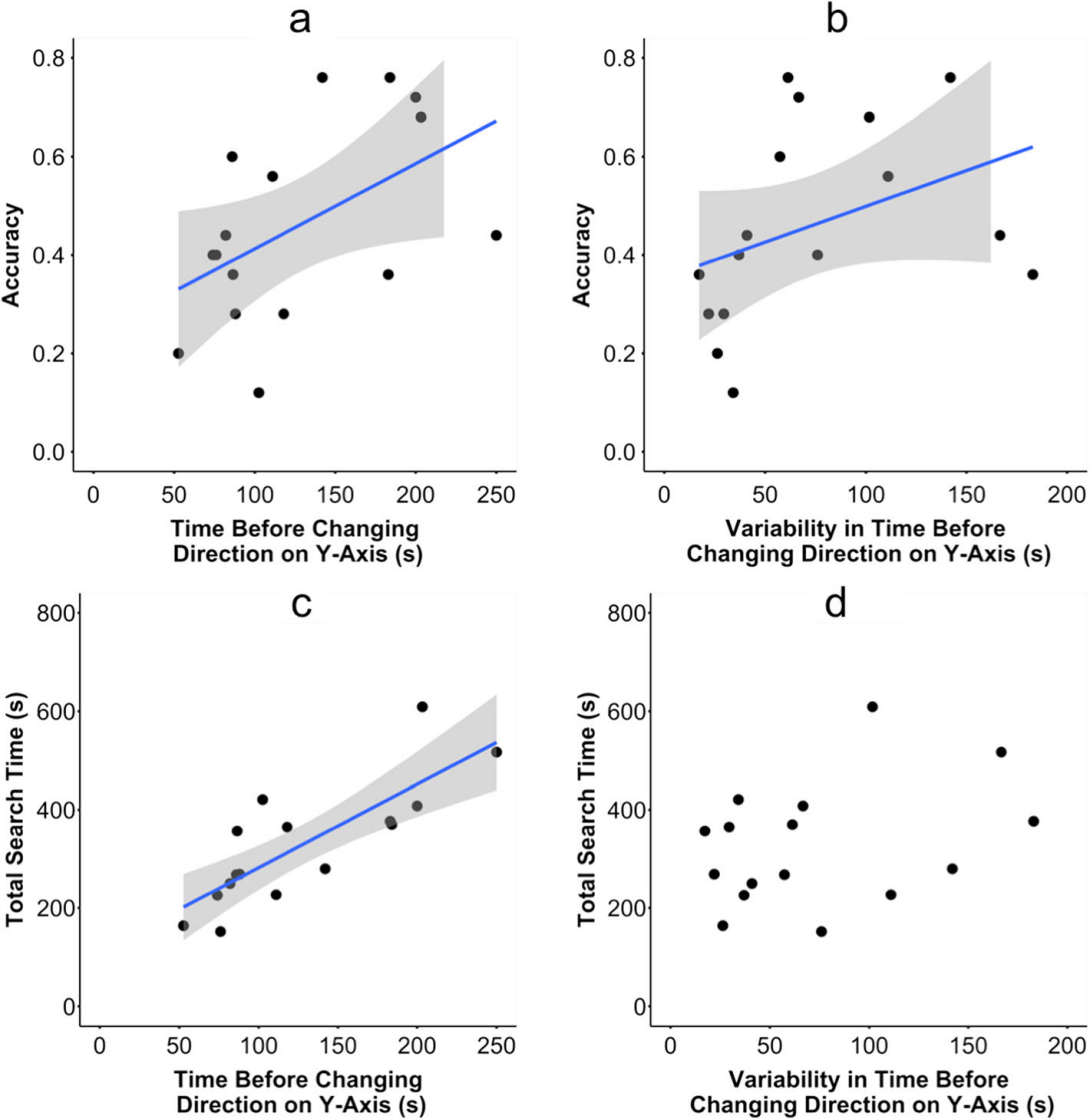
Search speed to predict Accuracy and Total Search Time for top-to-bottom-participants on the *y-axis*. On the basis of the Fourier analysis, we computed an approximation of participants’ modal time before changing direction in seconds (s) on each axis, using their dominant frequency component. By dividing the modal time before changing direction by the next most commonly occurring time before changing direction we computed the variability in time before changing direction in seconds (s). Accuracy was the proportion of coins detected and Total Search Time indicates the total amount of time spent searching in seconds (s). Figure 7 shows the modal time before changing direction (**a**) and the variability in time before changing direction (**b**) to predict Accuracy, plus the modal time before changing direction (**c**) and the variability in time before changing direction (**d**) to predict Total Search time. Note: *lines of fit* are shown when a statistically significant relationship is present

For the top-to-bottom group, there was a very strong trend for accuracy to be predicted by modal time before changing direction and variability in time before changing direction (β_1_ = 1.229, *F*(1,14) = 5.926, *p* = 0.029, adj R^2^ = 0.247; β_1_ = 1.982, *F*(1,14) = 5.716, *p* = 0.031, adj R^2^ = 0.239; see Fig. 7a and b). Participants were more accurate when they took longer before changing direction and varied their time before changing direction. Total search time was predicted by modal time before changing direction but not variability in time before changing direction (β_1_ = 0.956, *F*(1,14) = 27.21, *p* < 0.001, adj R^2^ = 0.636; β_1_ = 0.507, *F*(1,14) = 1.047, *p* = 0.324, adj R^2^ = 0.003; see Fig. 7c and d). Participants took longer overall to search when they took longer before changing direction. As predicted, increased accuracy was associated with slow search (i.e. longer before changing direction, i.e. turning), but surprisingly, it was variable search speed that was associated with increased accuracy rather than a consistent pace as predicted.

### Discussion

In Experiment 1a, we examined how searching for an unknown number of coins in an open space of grassland terrain was conducted. We predicted that accurate search would be reliant on: (1) a regular, systematic search strategy following a structured path (Dalmaijer et al., 2014; Donnelly et al., 1999; Gilchrist & Harvey, 2006; Keech & Resca, 2010); (2) narrow search corridors (Koopman, 1946, 1980); and (3) slow, consistently paced movement (Koopman, 1946, 1980).

The basic behavioral data showed the task to be extremely challenging, with task accuracy being at 45%. Many targets were missed despite participants taking a significant amount of time to explore the search area (on average, 7 min 33 s). Targets were, however, detected at a fixed rate throughout search. Furthermore, accuracy was predicted by the confidence ratings given by the participants, suggesting participants had some idea of how accurate they had been in the task.

The route maps showed participants tended to search using an ‘S’-shaped strategy, though sometimes embedded within a more complex pattern (see Fig. 3). This was confirmed in the analysis of data extracted using the Fourier analysis. Given that all participants exhibited regularity in their search path, we were unable to explore whether a regular search path predicted higher accuracy or not. However, the regularity of the ‘S’-shaped path made it possible for participants to define a search path that rarely contained crossovers and where the search width varied between 1 and 2 m. Following an ‘S’-shaped path minimized the memory demands inherent in the task relative to if a more irregular path was followed (Gilchrist et al., 2001). Presumably this width of search corridor adopted by participants was set according to their beliefs about the salience of targets in the context of the environment in which they were being sought. More or less salient targets would, respectively, lead to use of a wider or narrower search corridor.

Given that participants opted to search using a common search width, an important question is, how exhaustive is each participants’ search within their search corridor? Accuracy varied markedly across participants despite using the common search width and so using the common search width did not ensure that search was exhaustive. At least for some participants, the failure to search exhaustively was associated with a faster movement time reflected in the reduced modal time before turning. The implication of speeded search is that areas of the search corridor were left unexplored as forward body motion occurred at a rate too fast for the sweep of left-to-right head movements and associated eye movements. The fact that accuracy was associated with confidence is consistent with participants having some insight into the likelihood of the success or failure of their attempt at an exhaustive search (metacognitive awareness).

One might think of the relationship between time before changing direction and accuracy as reflecting a speed-accuracy trade-off but its observation is important. An observer tasked with ensuring or judging the quality of a search is unlikely to be able to make such a determination from the search path but it may follow from measuring differences in the time taken for search. Interestingly, slowed search distinguishes experts from novices in airport baggage-screening tasks (Biggs, Cain, Clark, Darling, & Mitroff, 2013). Calibrating how long a search task requires is, we suggest, a skill to be learnt both in complex visual searches and searches for targets placed in a more complex physical environment.

Variable search speed was also associated with increased accuracy, rather than a consistent pace, as predicted. This variability of search speed and accuracy found for the top-to-bottom participants was unexpected. On reflection, however, it is likely to be an effect associated with the task itself. Careful searchers slowed to ensure targets were clearly identified and marked as detected by the experimenters leading to variability in search speed as being identified with increased accuracy.

It is possible that the failure to find a relationship in the left-to-right group for time before changing direction and variability in time before changing direction for accuracy may be accounted for by the shorter time and distance between turns that participants had to make when moving left to right than top to bottom. Given that participants searching top to bottom were more accurate when they took longer before changing direction, the shorter distance before having to turn when searching left to right may have led to less efficient search. Measures based on participants movement through space may require sufficient movement time along axes, unfettered by the noise introduced by the slowing and speeding of turning itself, to become reliable indices of performance. In other words, the failure to find a relationship for accuracy for the participants moving left to right is likely to be a form of signal-to-noise problem.

## Experiment 1b

One concern is about the generality of the conclusions that can be drawn from Experiment 1a. It is possible that target conditions used may have forced a specific search strategy where participants searched along an ‘S’-shaped path for reasonably densely packed targets. The data were generated in response to a single grid and a fixed set of 25 targets. Within the limits of the search area, these 25 targets had a specific configuration and density. Many studies have shown that target prevalence influences the conduct of visual search (e.g. Fleck & Mitroff, 2007; Godwin et al., 2010b; Godwin, Menneer, Cave, & Donnelly, 2010a; Wolfe, Horowitz, & Kenner, 2005, Wolfe et al., 2007) and variations in target density are known to influence foraging (Cain et al., 2012; Wolfe, 2013). Might it be that the definition of search path and the width of the search corridors being searched are subject to change as target prevalence (along with target density and target configuration) varies? In Experiment 1b we repeated Experiment 1a, but had participants search three different search grids, each with a different number of targets present.

### Method

#### Participants

Fifteen participants (6 men and 9 women, mean age = 24.67 years, SD = 3.42) recruited from the University of Southampton community, with normal or corrected-to-normal color vision took part in the study for course credit. As in Experiment 1a, participants were screened to ensure visual acuity and normal color vision using Snellen’s (1862) chart for visual acuity and the Ishihara (1917) color plates.

#### Apparatus

The apparatus was the same as in Experiment 1a.

#### Stimuli

The grid size was the same as in Experiment 1a. In Experiment 1b, three grids were used with three different numbers of targets (5, 15, and 25 targets). As target prevalence changed, so did target density. Target density was, therefore, set at one target per 15 m^2^, one target per 5 m^2^ and one target per 3 m^2^. Experiment 1b was run in a different location and season to Experiment 1a and with a different depth of grass.

#### Procedure

The procedure was the same as in Experiment 1a, except all participants searched each of the three grid conditions. Condition order was controlled using a Latin Square design.

### Results

The data from Experiment 1b were analyzed in a similar manner to Experiment 1a in respect of accuracy, total search time, and confidence. The focus of Experiment 1b was the influence of target prevalence on the search path used by participants. It was not designed to explore variations in movement speed as target prevalence is likely to influence the distribution of movement speed and slowing associated with the detection of targets as reported in Experiment 1a. Analyses of movement speeds are, therefore, not reported.

#### Behavioral data

Basic measures of accuracy and total search time are presented in Table 1. On average, participants reported a confidence score of 7.13 on a ten-point scale for the five-target condition, 7.87 for the 15-target condition, and 8.47 for the 25-target condition. Accuracy, total search time, and confidence were compared across the three target prevalence conditions. A series of one-way ANOVAs revealed no effect of condition for accuracy (*F*(1, 14) = 0.392, *p* = 0.542, ges = 0.027) or total search time (*F*(1, 14) = 0.141, *p* = 0.713, ges = 0.01). Condition did reach significance for confidence (*F*(1, 14) = 32.941, *p* <0.001, ges = 0.702), with participants being more confident searching the 25-target condition than the five-target condition. Regressions exploring the relationship between confidence and accuracy in each of the target frequency conditions showed none to reach significance (*ps* > 0.807).

As in Experiment 1a, for each participant in each condition the time of finding each target was regressed against the ordinal number of that target as found by the participant. (i.e. 1st, 2nd, 3rd, etc.). The ranges of adjusted R-squared values were 0.733–0.995 (all *ps* < 0.238, the linear fits for five participants failed to reach significance), 0.84–0.992 (all *ps* < 0.002), and 0.923–0.993 (all *ps* < 0.001) for target prevalence of 5, 15, and 25, respectively. On average the first target was found after 5, 7, and 35 s in the 25, 15, and 5 target prevalence conditions, respectively. On average, the last target was found 13, 17, and 42 s before terminating search in the 25, 15, and 5 target prevalence conditions, respectively.

#### Search strategy

**Fig. 8:**
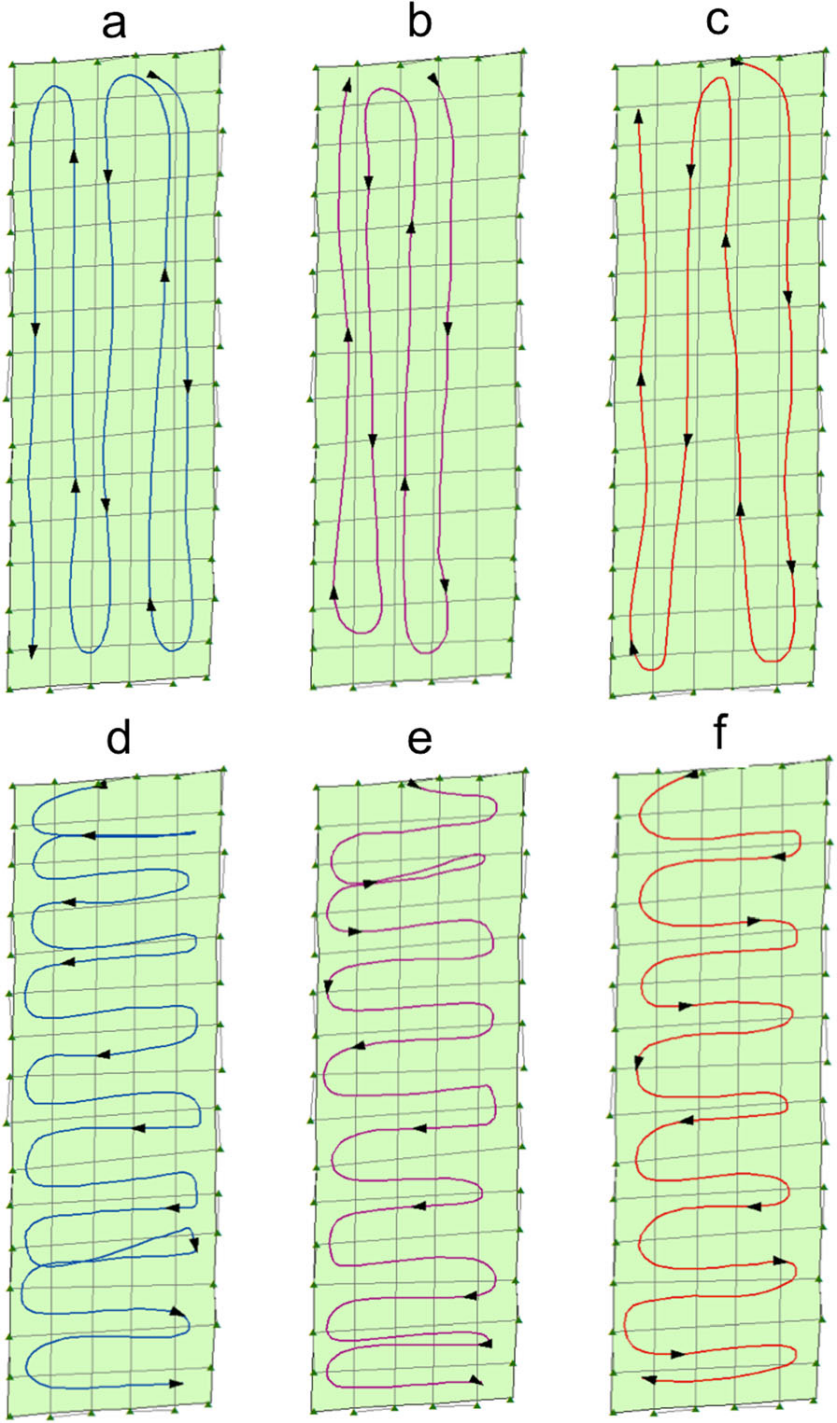
*Route maps* for Experiment 1b. Illustrations of examples of routes taken when searching. A participant using a common search strategy of top-to-bottom across target prevalence conditions (**a**, **b**, **c**) and a participant using a common search strategy of left-to-right across target prevalence conditions (**d**, **e**, **f**)

Of the 15 participants, technical difficulties led to two corrupted files such that complete data was available for 13 participants. Example route maps are shown in Fig. 8. Visual inspection of these route maps confirms the accuracy and search time data presented above, search strategy is mostly consistent across conditions. Of these 13 participants, seven searched top to bottom in all conditions and four searched left to right in all conditions. For these participants, there is no evidence of the route of the search paths changing with differences in target frequency.

### Discussion

Experiment 1b was easier for participants than Experiment 1a. The conditions of the experiment (both physical conditions on the ground and the fact that participants were better practiced) led to higher accuracy in Experiment 1b than 1a. Despite these differences, participants invariably adopted one strategy and then maintained it across conditions. The strategy chosen by participants was identical to that reported in Experiment 1a: an ‘S’-shaped path with a marginally higher likelihood for paths to go from top to bottom than left to right. A strategy that, irrespective of target prevalence, led targets to be detected at a consistent rate throughout the period of searching and with similar levels of success.^1^ These data are consistent with a view that, at least within the limits of the target prevalence tested in the present experiment, the search paths for searching for targets in open ground are determined in a manner independent of target prevalence.

While the search path is determined independent of target prevalence, the confidence with which search is conducted was not independent of target prevalence. Confidence in search reduced with reduced target prevalence. While participants were not told how many targets were in each grid, confidence may have been lowered by the experience of finding fewer targets in the five-target condition relative to the experience of finding an increased number of targets in the 25-target condition. Whatever the underlying cause of the reduced confidence in search when target prevalence was low, an important point is that, as in Experiment 1a, the lowered confidence of participants did not change the search path. Furthermore, Experiment 1b confirms that the search path followed in Experiment 1a was not a result of the relatively densely packed targets but holds over much more sparse target densities.

## Experiment 2

The coin detection task conducted in Experiments 1a and 1b was motivated by tasks such as police searching for clues to crimes in open grassland and soldiers searching for IEDs in theater. In everyday life, such tasks are rarely performed by individuals working alone, but instead by searchers working in teams. Two strategies for team working can be used. Search can be split across the search space or checked through sequential independent searches. Splitting search across participants might seem obviously advantageous. Doing so can minimize load allowing faster searching to occur (Brennan, Chen, Dickinson, Neider, & Zelinsky, 2008).

However, the evidence from Experiments 1a and 1b suggests that the approach to searching across participants might not translate into improved search accuracy when splitting search across space. The failure to find a relationship between width of the search corridor and accuracy implies that being seen to adopt an ‘S’-shaped search path should not translate into beliefs about equivalent search competence. Put another way, splitting search by area allows the risk of a poor searcher appearing competent but working unchecked and a risk of coordinating search effort (Brennan et al., 2008).

A better strategy might be for pairs of searchers (henceforth dyads) to benefit from the independent probability summation that occurs when searching independently across search spaces. Independent probability summation is the summed probability of detecting each target that comes from the addition of each searcher’s likelihood.

In Experiment 2, we repeated Experiment 1a but with dyads searching for coin targets. They were allowed to define their own search strategy and were free to communicate in whatever way they felt appropriate. Dyads electing to split search by area and those conducting independent search were defined post-hoc. The performance of pairs electing to split search by area and those electing to not do so was compared, as was the performance of both groups to the individuals reported in Experiment 1. We predicted that if individuals in the dyad worked independently of each other (i.e. search effort doubled over the entire space), then accuracy would be higher as performance would benefit from the summation of two searchers, without the risk of coordination attempts. This increased accuracy will come at the cost of increased time relative to those who split search by area. Finally, we also predicted that as in Experiment 1a and 1b, participants would search systematically using an ‘S’-shaped route searching primarily either top to bottom or left to right.

### Method

#### Participants

Thirty-four participants (14 men and 20 women, mean age = 26.38 years, SD = 5.79) recruited from the University of Southampton community, with normal or corrected-to-normal color vision took part in the study for course credit. As in Experiment 1, participants were screened to ensure visual acuity and normal color vision using Snellen’s (1862) chart for visual acuity and the Ishihara (1917) color plates. Participants were allocated into 17 dyads.

#### Apparatus

To ensure comparability across Experiment 1a and 2, both members of the dyad held a staff as they searched. The Total Station theodolite was, however, limited to recording coordinates for one participant in each dyad. We did attempt to record similar data from the second member of each dyad using a satellite-based system but this proved unreliable and so these data were unfortunately not viable for our analyses.

#### Stimuli

The experiment was conducted on the same areas of grassland as in Experiment 1a and at the same time of year. The grid size was also the same and the same 25 UK sterling two pence coins were used in the same grid cell positions.

#### Procedure

The procedure was the same as in Experiment 1a, but with participants working in dyads.

### Results

In Experiment 2, all dyads detected coins and so no dyads were removed from analysis.

#### Behavioral data

Considering dyads as a single group, basic measures of accuracy (total for the dyad) and total search time are presented in Table 1, alongside the individual’s data from Experiments 1a and 1b. On average, dyads detected 68% of targets and spent just over 6 min searching. On average, participants reported a confidence score of 7.12 on a ten-point scale. To assess the advantage of dyad search over individual search, the data from Experiment 2 were compared to those from Experiment 1. Independent t tests revealed dyads searched more accurately than individuals (*t*(42.757) = 4.089, *p* < 0.001) but that there was no difference in search time (*t*(34.374) = –0.89, *p* = 0.19). Note, for the dyads, search time is defined as the time at which both members of the dyad agreed search had finished, rather than the summed time of the two searchers. Dyads were more confident in their search performance than individuals *t*(36.438) = 1.75, *p* = 0.04) though unlike the individuals in Experiment 1a, confidence ratings for the dyads failed to predict accuracy (β_1_ = 0.043, *F*(1,15) = 2.291, *p* = 0.151, adj R^2^ = 0.07).

#### Shared searching

To explore how participants in each dyad split their search, for each dyad, targets were classified as detected by: (1) participant one; (2) participant two; (3) both participants; or (4) missed by both participants (see Table 2 for means).

**Table 2:** Shared searching data

Sole hits	Joint hits	Misses
12.29 (4.81)	5.35 (5.56)	7.35 (2.8)

The mean number of targets detected by only one participant in a dyad (Sole hits), the number of targets detected by both participants in a dyad (Joint hits) and the number of targets missed by both participants in a dyad (Misses)

Parentheses indicate Standard Deviation

These data were used to classify, for each dyad, the number of misses, individual hits, and joint hits. The number of joint hits was subtracted from the sum of the number of individual hits from each dyad and divided by the total number of hits. This gives a ratio from +1 (totally independent) to –1 (totally shared) to give a measure of target detection strategy that is independent of accuracy.

To test whether the search style adopted by the dyad influenced accuracy, the dyads were then split into two groups: (1) dyads with a split search ratio higher than 0, meaning they tended to search independently (*n* = 5); and (2) dyads with a split search ratio lower than 0, meaning they tended to split search between them (*n* = 12).

**Fig. 9:**
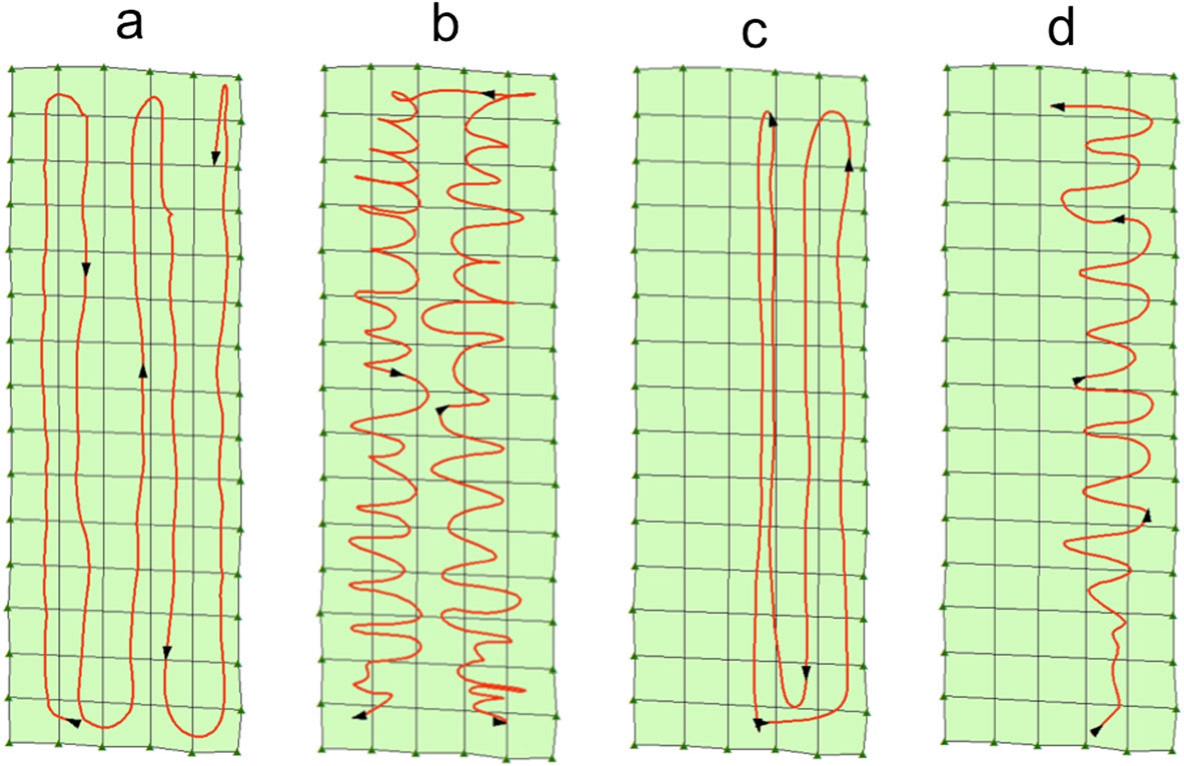
*Route maps* for Experiment 2. Illustrations of examples of routes taken by independent searchers searching top to bottom (**a**) or left to right (**b**) and split searchers searching top to bottom (**c**) or left to right (**d**)

The data from the Theodolite system provided data to analyze the search strategy from one member of each dyad and we used the route maps to seek direct evidence that some dyads tended to split search. In theory, this gave us a total of 17 participants; however, due to technical faults, two participants could not be included in the search strategy data, giving a total of 15 participants. Inspection of the route maps from the individual within each dyad for which movement data were recorded provides evidence consistent with splitting the dyads into shared and independent searchers (see Fig. 9). These route maps show independent searchers tend to inspect the whole search area whereas split searchers tend to inspect a sub-area of the search space. Of the five independent searchers, two searched top to bottom, three searched left to right and one performed a hybrid search strategy. Of the nine split searchers, four searched top to bottom and five searched left to right.

Both dyad groups, whether tending to search independently or splitting search, were significantly more accurate than individual searchers (*t*(11.479) = 5.36, *p* < 0.001 and *t*(35.087) = 2.885, *p* = 0.007, respectively). A final t-test confirmed that dyads tending to search independently were more accurate than those tending to split search (*t*(8.699) = 3.053, *p* = 0.014). Dyads who tended to split search took significantly less time to search than individuals (*t*(37.824) = –2.83, *p* = 0.007) but there was no significant difference between dyads who tended to search independently and individuals (*t*(5.162) = 1.079, *p* = 0.328). The difference in search time between dyads who tended to search independently and dyads who tended to split search failed to reach significance (*t*(4.389) = 2.315, *p* = 0.076).

#### Search strategy

The movement data recorded from the individual within each dyad do allow us to explore one other issue (note that computation of search width and search speed makes no sense as the data come from only one member of each dyad). As in Experiments 1a and 1b, each participant can be classified as searching primarily top to bottom or left to right or using a hybrid strategy (see Fig. 9). Six participants used a top-to-bottom strategy (two independent and four split) and eight (three independent and five split) used a left-to-right strategy. Using a Fisher’s exact test and comparing these data against those from Experiment 1 (where across 1a and 1b, 23 used a top-to-bottom strategy and 16 used a left-to-right strategy), revealed a non-significant result (*p* = 0.358). Working in dyads did not significantly change the likelihood of direction of searching.

### Discussion

The results of Experiment 2 confirm the results of Experiment 1a in showing the coin detection task to be very demanding. While accuracy was higher in Experiment 2 than 1a, performance was still not at ceiling for any pair. More importantly, Experiment 2 showed two critical results. First, dyads searched more accurately than individuals searching the whole space alone. They did this whichever strategy they adopted, whether splitting search or searching independently of each other. Whatever the cost of coordinating search effort (Brennan et al., 2008) across the open area of grassland, these costs are overcome by dyads. The benefit to search accuracy of dyadic relative to individual searching comes with no change in search time (when both dyad groups are considered together). Within the same unit of time available, dyadic search benefitted from twice the resource being applied to search. Second, and critically, as predicted search performance was best when dyads searched independently rather than when splitting search. Dyad accuracy benefited from the summing of the performance of two independent searchers rather than splitting search. Furthermore, as in Experiment 1a and 1b and from the data available, it seems participants searched systematically using an ‘S’-shaped route searching primarily either top to bottom or left to right.

We conclude that, at least for the experimental conditions used in Experiments 1a and 2, search is more accurate when carried out by dyads over individuals, but that dyads are most accurate when providing two independent passes over the open space rather than when trying to split search such that overall performance benefits from independent probability summation.

## General discussion

The present study set out to explore how participants search for targets on open grassland. The task reflects a class of search problem that has received little attention in psychology, but is one which is a common problem in a wide range of security-critical and evidence-gathering scenarios. Participants searched open grassland, either individually (Experiments 1a and b) or in dyads (Experiment 2), for an unknown number of small targets of low salience (coins). Significant numbers of misses were made across all experiments indicating that although possible, all versions of the task were difficult.

Experiment 1a revealed individuals typically use an ‘S’-shaped search strategy with a common search width of between 1 and 2 m and with targets being found throughout the search time. Furthermore, when searching from top to bottom, increased accuracy is associated with slow search (i.e. longer time before changing direction) but with variable speeds (i.e. stopping to check targets). For all participants, targets were detected at an even rate throughout searching. The simplest account we offer of these data is that task performance was not affected by reducing vigilance over the time course of searching. This basic account would lead to a linear fit between the ordinal number of targets and the detection time of targets.

It is possible that the gradient of the linear relationship between the ordinal number of targets and detection time is influenced by the increasing cost of searched locations and found targets as search progresses or, alternatively, by the increasing efficiency of perceptual processes as more targets of the same type are discovered (Cain & Mitroff, 2013). We cannot exclude either possibility as contributing to the rate of target detection. However, the ‘S’-shaped strategy seems to us to be followed to minimize the memory load of the task at the same time as maximizing the probability of an exhaustive search (Gilchrist et al., 2001).

It may be the case that the ‘S’-shaped strategy is a function of the shape and area of the search region. While the 75 m^2^ search area was much larger than that typically used in visual search studies, it was still a regular rectangle and defined in a manner that allowed planning (i.e. could be seen as one and defined by cones). Further studies should explore how search strategy is influenced by the regularity, shape, and size of the search area. In addition, the manner with which the search area is defined by landmarks that remain visible from all points of the search area may be important. Understanding how search strategy changes across types of search area will be important as, in the real world, areas being searched may well vary in size and shape.

Experiment 1b revealed that the accuracy, total search time, ‘S’-shaped strategy and the even rate of target detection across the duration of searching are unchanged by varying target prevalence. We view this evidence as important in as much as it shows that the specific conditions of Experiment 1a did not lead to an artefactual result, but instead one that generalizes beyond one set of conditions. It is, of course, the case that in Experiment 1b the same participants searched in all conditions. As such, they will have experienced search for targets occurring at different prevalence rates in a reasonably short period of time. For this reason, we would not claim that these data definitively demonstrate that target prevalence does not influence search strategy in this task. For that conclusion to hold, more extensive experimentation would need to be conducted, perhaps using a blocked design and even lower levels of target prevalence.

The existence of a structured approach to searching for coins is important. Structured search allows the possibility individuals within dyads might coordinate search strategies. Coordinating search by splitting search across space might have reduced total search time if not also improved accuracy. In fact, Experiment 2 revealed dyads were more accurate than those searching individually, even when splitting search over space.

Experiment 2 showed that the most effective strategy for dyads was to conduct two independent searches rather than splitting search. It is important to note that conducting independent searches is not a failure to coordinate search across dyads. It is possible that dyads held an implicit understanding that there are risks and costs associated with the coordination of split search (i.e. even if a partner’s search appears to be conducted in a serious manner, it does not necessarily predict good target detection). These costs lead to worse performance than can be achieved through summing the independent probabilities for target detection. The clear conclusion when searching for threat critical targets is that searching for targets in open ground is enhanced by dyads working in tandem but searching independently. Of course, how this conclusion stands up as search teams that go beyond dyads, and as the search area increases beyond that used in the present experiments, is uncertain.

It is important to note that in the present study, the tendency to opt for either a split search or independent search strategy reflected a decision made within the dyad. Decisions about strategy might be open to review throughout the course of conducting search tasks if, for example, a partner appeared to be searching without due care and attention. There is evidence that humans can make attributions about others from observing motor control during task performance (Wolpert, Doya, & Kawato, 2003). The data from Experiment 1a do suggest, however, that searchers should be wary of believing search is being conducted accurately simply by observing the strategy adopted by partners and the time taken to search.

In a related manner, the relative influence of social facilitation (Triplett, 1898; see also Strauss, 2002) and social loafing (Karau & Williams, 1993) may also raise or diminish overall performance. In the case of the present study, comparison across Experiment 1a and 2 shows social facilitation tended to improve performance by raising accuracy without changing search time. Of course, what influences decisions within the dyad to work together in one mode or another may reflect multiple factors. Low levels of willingness to trust and high levels of thoroughness may both be reflected in high levels of independent searching of the whole search space. There is evidence that differences in levels of individuals’ extraversion and agreeableness, and overall conscientiousness of pairs, influences the likelihood of individuals attributing challenges to paired working (Bono, Boles, Judge, & Lauver, 2002). The present study does not provide data to help understand the role of these mediating variables in predicting dyadic search strategy. Nevertheless, it should be acknowledged that extraversion, agreeableness, and conscientiousness may predict the likelihood of individuals conducting a systematic search across open space, and the strategy of search pairs. It is an issue worthy of future study.

The nature of the search task is also likely to influence the decision-making and performance of the dyads. Although the present task was a first effort to understand the detection of clues in crime scenes and IED detections in war zones, it is, of course, far removed from both situations. It is important to emphasize that there is a very significant gap between our experiments and the situations that motivated them. We were interested in understanding how effectively search areas are explored when search must be exhaustive. We deemed this an important question because ensuring the exhaustiveness of search remains a requirement even when finding clues or IEDs. That said, in the present study, the implications for failing to find coins was simply reduced accuracy in relation to performance. In this sense, we can only be sure that our conclusions hold for the limited case of this benign task performed in the conditions explored in this present study. In addition to the need to explore how search is affected by changes in the size and shape of the search grid and the uniformity and prevalence of targets, we must also be alert to the fact that changes in strategy will almost certainly occur based on the consequence of a miss or a failure to search exhaustively in the task. We consider it important to emphasize this final point.

## Conclusion

In conclusion, Experiment 1a revealed the importance of systematic search for target detection, with participants using an ‘S’-shaped search route with a common width of search corridor. Participants found targets at a common rate throughout searching along the search route. When searching top to bottom, increased accuracy was associated with slower searching. Accurate target detection occurred when time allowed for eye, head, and body movements to be made to search across the width of the search corridor. Experiment 1b revealed that variations in target prevalence did not change the shape of the search route followed by participants, nor the overall accuracy or time spent searching. It did, however, influence the confidence in their search performance with confidence lowered when targets were relatively infrequent. Experiment 2 showed how dyads improve target detection relative to when search is conducted by individuals. This is true when splitting search but especially when improved accuracy results from the summed total of conducting two independent searches.

The present study has outlined a number of important findings, methods, and analyses in relation to searching for targets in open space. Future studies should seek to show how the search strategies that we have outlined generalize or are modified by stimulus and environmental conditions. For example, whether systematicity survives under conditions of very low target prevalence (Godwin, Menneer, Cave & Donnelly, 2010a) or target absence (Schwark, MacDonald, Sandry, & Dolgov, 2013), changes in the size and shape of the search area (Smith et al., 2008), variations in target type and identity (Menneer, Barrett, Phillips, Donnelly, & Cave, 2007), and the presence of distraction and the possibility of concealment (Godwin, Liversedge, et al., 2015). Furthermore, how dyadic working influences the effectiveness of these strategies in enhancing target detection for multiple targets placed in open ground. In particular, how the dyadic performance is changed by explicit instruction to follow specific strategies, time pressure, and perceived risk.

## Abbreviations

ANOVA: Analysis of Variance
ESW: Effective search width
IED: Improvised explosive device
SD: Standard Deviation

## Footnotes

1 It is true that a number of participants in the five-target prevalence condition did not have a significant linear fit between the number of the target detected and time. However, there are many fewer data points in this condition than in the 15-target and 25-target prevalence conditions. As such, less reliability should be expected. Regardless, the general trend of these data is linear when considering all participants.
